# Interpreting Microaggression as a Determinant of Wellbeing

**DOI:** 10.1007/s40615-022-01426-z

**Published:** 2022-10-12

**Authors:** Adekunle Adedeji, Tosin Tunrayo Olonisakin, Franka Metzner, Johanna Buchcik, Wandile Tsabedze, Klaus Boehnke, Erhabor S. Idemudia

**Affiliations:** 1 Faculty of Humanities, North-West University, Mafikeng, South Africa; 2 Faculty of Life Sciences, Hamburg University of Applied Sciences, Hamburg, Germany; 3 Department of Medical Psychology, University Medical Center Hamburg-Eppendorf, Hamburg, Germany; 4 Bremen International Graduate School of Social Sciences (BIGSSS), Jacobs University Bremen, Bremen, Germany

**Keywords:** Microaggression, Inequality, Wellbeing, Cultural identity, Racial identity, Microinsults, Microassaults, Invalidations

## Abstract

Despite the increasing interest in exploring microaggression in the humanitarian context, there remains uncertainty on its mechanism for affecting life outcomes. There is a lack of studies on ethnic and racial minorities in non-western countries. The current research explores dimensions and manifestations of microaggression and how they affect wellbeing in a multicultural setting. The study uses a qualitative approach with 15 focus group discussions (FGDs) and 66 participants conducted in 4 provinces of South Africa: Gauteng (*k* = 6), North-West (*k* = 3), KwaZulu-Natal (*k* = 3), and Western Cape (*k* = 3). The recorded FGDs were transcribed using the intelligent verbatim technique. The transcripts were then analysed using a phenomenological approach. Data analysis was done stepwise using the deductive coding technique. Results show that participants’ perception of the dimensions of microaggression varies depending on the manifestation as verbal, behavioural, or systemic. Furthermore, variations in patterns and reactions to dimensions of microaggression were linked with participants’ racial identity. It further confirms that experiencing discrimination is associated with poorer wellbeing. Connectedness to the ingroup provides stability and certainty in multi-group societies due to the group rivalry that pervades such societies.

**Supplementary Information:**

The online version contains supplementary material available at 10.1007/s40615-022-01426-z.

## Background

Exploring microaggression as a social construct has continued to gain relevance in humanitarian and social discourse. Several studies in health care [1, 12, 16] have proposed that daily experiences characterised by aversive discrimination may have significantly more influence on wellbeing and self-esteem than traditional overt forms of discrimination [31, 44]. However, despite the increasing interest in exploring microaggression in the humanitarian context, there remains uncertainty on its mechanism for affecting life outcomes. This uncertainty emphasises the challenge of describing, measuring, and interpreting microaggression as a form of discrimination via “aversive racism” and/or “implicit bias.” Furthermore, it highlights the still limited scientific understanding of microaggression and its predictive feature for health and other life outcomes.

Microaggression is referred to as brief and commonplace daily indignities — whether intentional or unintentional — that communicate hostile, derogatory, or harmful slights and insults to the target person or group [26, 47, 48, 50]. Previous studies have attempted to categorise microaggression to ease the exploration of this social construct. Sue et al. [47] identified three dimensions of microaggressions — microinsults, microassaults, and invalidations — assumed to differ in the intensity and pattern in which they are experienced. Further exploration of microaggression also submits that it may be verbal, behavioural, or systemic [26, 50]. Systemic experiences of microaggression are subtle discrimination that occurs within society. This systemic manifestation is the same as what Sue et al. [47] referred to as environmental. They are social norms that create settings or situations that assail individual identity.

The current study adopts the categorisation of microaggression as microinsults, microassaults, and microinvalidations and its manifestations as verbal, behavioural, and systemic. In doing so, microinsult is referred to as behaviours, verbal exchanges, or systemic patterns based on negative stereotypes that unintentionally discriminate against a person or group [47]. Like other dimensions, microinsults transmit insensitive and disparaging messages to a person’s cultural identity or background, albeit with no intention to hurt. On the other hand, microassaults are explicit behavioural, verbal, or systemic acts of discrimination that communicate that the targeted party is of lesser worth. They are characterised primarily by attacks meant to hurt the target through name-calling, avoidant behaviour, or purposeful discriminatory actions [47]. Unlike microinsult, microassault is intended to stigmatise an individual or members of a targeted group. The third category, invalidation, describes behaviours, verbal exchanges, or systemic patterns that negate, neutralise, or deny unique cultural or racial experiences [47]. Like microinsult, microinvalidation is often not intended to hurt or harm.

As global and national demographics continue to react to migration trends, the implications of microaggression and the need for cultural and racial harmony for better life outcomes become more pronounced. This is more so the case as culturally homogenous towns and cities adopt multiculturalism [9, 10, 49]. Furthermore, recent studies have emphasised the role of social and community integration for improved life outcomes in multicultural settings [4, 8, 46]. Consequently, the experience of discrimination hinders positive life outcomes, such as life satisfaction, health, and wellbeing [23, 41, 53]. Despite this evidence and the potential implication of microaggression for life outcomes, few research projects have explicitly investigated the role and mechanism of microaggression in its varied dimensions and manifestations in relation to wellbeing and other life outcomes.

In a survey of 152 Asian Americans, Ong et al. [40] measured somatic symptoms and racial microaggressions for 14 consecutive days. They found that 78% of participants reported some form of racial microaggression [40]. Furthermore, they reported elevations in daily microaggressions and that greater microaggressions predicted somatic symptoms and negative affect [40]. Another empirical investigation found that racial microaggression was significantly and positively associated with cultural mistrust and inversely related to wellbeing [29]. Other studies also found that microaggressions can have harmful physical and emotional effects on people of a specific group [2, 24, 39].

Moreover, Lui and Quezada [32] examined microaggression as a form of stressor that negatively affects people with marginalised statuses. In a meta-analytic and narrative review using 72 independent study samples (*n* = 18,718), the authors found a statistically significant summary correlation between microaggression and different adjustment outcomes such as internalising problems, stress/negative affect, and positive affect/adjustment [32]. A recently published scoping review by Crawford et al. [15] even showed microaggression as an experience of racism related to perinatal mental health outcomes like postpartum depression or anxiety.

However, these explorations lack an in-depth understanding of the dynamics of categorises and manifestations of microaggression. For example, the difference between microinsults, microassaults, and microinvalidation and the differences between verbal, behavioural, and systemic manifestations of the dimensions of microaggression has not been sufficiently considered. These differences cannot be dismissed as they remain harmful to wellbeing, self-esteem, public health, and other life outcomes. Therefore, understanding the unique impact of individual dimensions and manifestations could be instrumental in facilitating positive life outcomes. The meta-analytic review of Lui and Quezeda [32] showed that very few studies tested whether microaggression predicted adjustment outcomes above and beyond overt discrimination and individual difference factors (e.g. gender, racial, and health status) or examined the indirect mechanisms that may link microaggression to adjustment outcomes. Similarly, in a literature review, Wong et al. [50] found that most of the existing literature on racial microaggressions has focused on the experiences of individuals of African, Asian, and Latino origins in western countries but found limited studies on ethnic and racial minorities in non-western countries.

South Africa, often referred to as a rainbow nation because of its multicultural, multiethnic constituency [3], is one of the most culturally and racially diverse countries. Racial and cultural identities in South Africa have historically influenced the structure of social, economic, and political spheres [28]. This is attributed to the segregation in Apartheid South Africa [34], in which Black, White, Coloured, and Indian racial categories were created to enforce physical and social segregation [14, 35].

Racial classification in South Africa placed individuals in one of four groups: “Whites,” “Blacks” (native African), “Indians,” and “Coloured” (people of mixed race). Despite the Apartheid rule being terminated in 1994, these racial categories remain relevant in political, social, and economic pursuits in South Africa. For example, the “Broad-Based Black Economic Empowerment” (BBBEE) by the South African government racially categorised South Africans and applied a system of incentives across government and the private sector.

These racial classifications remain relevant for evaluating South Africa’s social wellbeing performance and as a measure of social equality. The cultural and racial discord in South Africa is attributed to unequal access to socioeconomic resources among the different racial groups [5, 22]. The diverse cultural attributes and the clear racial divide [22] make South Africa ideal for exploring different features and correlates of microaggression. Different social survey reports attest to the distrust, mutual suspicion, and antagonism between the racial groups in the country [25, 37]. Thus, microaggression toward racial outgroups is a readily observable feature of South African society.

### Study Objectives

**Fig. 1:**
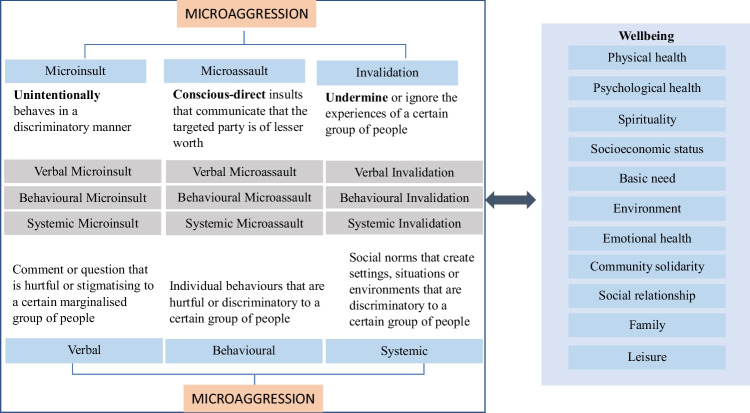
Conceptual framework of the dimensions and manifestations of microaggression and their association with wellbeing adapted from Sue et al. [47]

The current study explores the dimensions and manifestations of microaggression and how they affect wellbeing in a multicultural setting using the example of South Africa (see Fig. 1). Pursuing this line of research will explain how social interactions and expectations affect life outcomes for different groups. The following objectives were set:

1. To examine the dimensions of microaggression by racial identity,
2. To explore racial differences in the perceived effect of microaggression and its dimensions and manifestations on wellbeing.

### Conceptual Framework

As depicted in Fig. 1 below, the relationships between microaggression and wellbeing are complex and multidimensional. To further explore these associations, we expanded the framework presented by Sue et al. [47] to link different manifestations and dimensions of microaggression to different wellbeing outcomes. In doing so, nine subconstructs of microaggression were conceptualised:

1. Verbal microinsults — statements, comments, or questions from people that unintentionally stigmatise or hurt an individual based on their social or cultural identity.
2. Behavioural microinsults — behaviours from people that unintentionally stigmatise or hurt an individual based on their social or cultural identity.
3. Systemic microinsults — negative beliefs or environmental factors that are socially considered normal about a group that unintentionally stigmatises or hurts an individual that identifies with that group.
4. Verbal microassault — statements, comments, or questions from people intended to communicate that an individual based on their social or cultural identity is of lesser worth.
5. Behavioural microassault — explicit behaviours from people intentionally communicating that an individual based on their social or cultural identity is of lesser worth.
6. Systemic microassault — negative beliefs and social norms that create settings or situations that intentionally assail individual identity or communicate that people of a group are of lesser worth.
7. Verbal invalidation — statements, comments or questions from people that belittle, trivialise, or deny a group’s unique experiences.
8. Behavioural invalidation is behaviours from people that unintentionally belittle, trivialise, or deny a group’s unique experiences.
9. Systemic invalidation — social norms that create settings, situations, or environments that unintentionally assail individual identity.

We hypothesise that each of these complex manifestations and dimensions of microaggression uniquely affects subjective wellbeing. Subjective wellbeing refers to how people experience and evaluate different aspects of their lives [17]. It measures overall life satisfaction and happiness and has become increasingly common in psychological, social, and humanitarian research. The experience of microaggression among the current sample is assumed to influence the subjective evaluation of wellbeing.

## Method

### Study Design

This study uses a qualitative approach to explore the experience of microaggression and its implication for wellbeing. Fifteen focus group discussions (FGDs) were conducted in four provinces in South Africa. The flexibility and adaptability of the FGDs support the subjective evaluation of microaggression and in-depth exploration of its implication for wellbeing [13, 33].

### Procedure

**Fig. 2:**
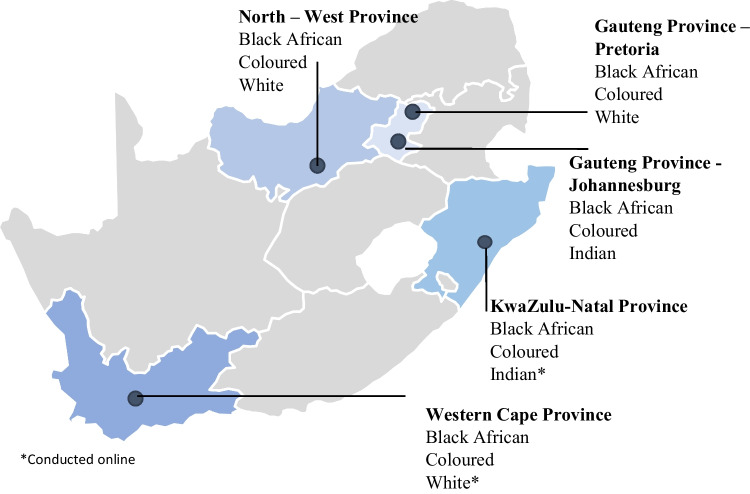
Location for focus group discussions by the province of residence in South Africa and participants’ racial identity (This figure features in a different publication on the conceptualisation of wellbeing by Adedeji et al. [6]

The FGDs were conducted in Gauteng (*k* = 6), North-West (*k* = 3), KwaZulu-Natal (*k* = 3), and Western Cape (*k* = 3) provinces in South Africa (Fig2). This selection was based on the geographical positioning (i.e., North, West, South, and East) and the unique demographic characteristics such as population density and the racial composite of these provinces.

Recruitment information was disseminated through personal contacts, project website, flyers, and social media (e.g., Facebook and WhatsApp). Purposive sampling was used to ensure the participants were from various demographic and socioeconomic categories. A paper–pencil questionnaire was distributed to participants at the beginning of the FGDs. This questionnaire was used to collect data on participants’ demographic characteristics, i.e., age, gender, racial identity, and socioeconomic status, based on yearly income and education. Furthermore, each participant was asked to provide a list of things that were essential to their wellbeing in everyday life. These lists were later referenced to discuss the effect of microaggression on individually defined wellbeing.

The FGDs were held between June and December 2021; further information on the study implementation can be found in Adedeji et al. [6]. The 15 FGDs were moderated by 2 persons. AA (male) moderated 13 FGDs, while the remaining two FGDs were moderated by TO (female). Both moderators were trained in social research. There was an average of 5 participants per discussion (range 3–9 participants). Each FGD lasted an average of 1 h and 45 min (range 56 min to 2 h 10 min). Each participant was asked to state factors that contribute to their wellbeing following the Ravens-Sieberer et al. [42] (i.e., “Thinking about your life more completely, what are the things that make you feel well?”). Furthermore, participants were inquired to describe their perception and experience of the different dimensions (microinsults, microassaults, microinvalidations) and manifestations (verbal, behaviour, and systemic) of microaggression. Each of the dimensions was related to the manifestation in sequential order, i.e., verbal microinsults, behavioural microinsults, and systemic microinsults. Afterwards, participants using a three-point scale (1 = least impactful, 2 = moderately impactful, 3 = most impactful) were asked to rank the three dimensions of microaggression regarding their differential effects on wellbeing (e.g. “How would you rank in descending order microinsult, microassault, and invalidation in reference to their effects on your wellbeing?”). Likewise, they were further asked to rank the three manifestations of microaggression concerning their effects on wellbeing (e.g. “How would you rank in descending order verbal, behavioural, and systemic microaggression in relation to their effects on your wellbeing?”).

The moderator explained each concept and presented multiple examples to help the participants better understand and differentiate between the different dimensions and manifestations of microaggression (see Appendix A1). Follow-up questions were asked to explore the subjective implications or effects of microaggression on the highlighted predictors of wellbeing (e.g., How would you say these experiences influence your life outcome?) [11].

### Participants Characteristics

Sixty-six South African adults (aged 20 to 71, *M*_age_ = 35.7 years, SD = 13.0) participated in the FGDs and completed the survey questionnaire. Half of the participants were females (*n* = 33), while 32 were males, and one reported the “other” gender category. Furthermore, Black South Africans represent 39% (*n* = 26), Coloureds 32% (*n* = 21), Whites 21% (*n* = 14), and Indians 8% (*n* = 5). Similarly, more than half of the participants were from Gauteng (33%) and North-West (33%) provinces. About 18% were from KwaZulu-Natal, while 15% were from Western Cape. Socioeconomic data using the Bureau Market Research (BMR) income classification system (2016) showed that 20 participants were poor. Similarly, 15 were in the low emerging middle class, 15 in the emerging middle class, 8 in the realised middle class, and 8 in the upper middle class. On the other hand, data on education shows that 40 participants had at least a tertiary education. In addition, 17 had a high school leaving certificate (Matric or equivalent), 7 had a secondary school education, 1 had a primary school education, and 1 had no formal education.

### Data Analysis

The recorded FGDs were transcribed using the intelligent verbatim technique [51]. Participants were coded based on their province and racial identity (e.g., NWBLK represents North West, Black South African); these codes are referenced in the result section. The transcripts were then analysed using a descriptive phenomenological approach. This approach is particularly relevant to understand the most essential meaning of a phenomenon of interest from the perspective of those directly involved in it [21]. Data analysis was performed stepwise using the deductive coding technique [30]. The first coding was used to classify dimensions and the manifestation of microaggression and how they differ based on racial identity. Participants’ subjective evaluation of wellbeing was categorised into 11 subconstructs. These are physical health, psychological health, emotional health, social relationship, family, spirituality, basic need, leisure, community solidarity, environment, and socioeconomic status.

Further qualitative analyses were done to explore the interpretation of dimensions of microaggression (microinsults, microassaults, invalidations) and their effects on participants’ subjective wellbeing based on racial identity. Then, axial and selective coding was used to interpret the manifestation of microaggression (verbal, behaviour, and systemic) for the implication for wellbeing. These codes consisted of short sentences or single words, for example, “strong effect on the socioeconomic determinant of wellbeing.” Results from the ranking were averaged based on the number of participants from the same racial group. For example, the individual ranking of dimensions of microaggression by Black South African participants was summed up and divided by the number of Black South African participants.

## Results

Results from axial and selective coding reveal that participants’ perception of the dimensions of microaggression varies depending on the manifestation as verbal, behaviour, or systemic. Furthermore, further variation in patterns and reactions to dimensions of microaggression was linked with participants’ racial identity.

### Dimensions of Microaggression by Racial Identity

**Fig. 3:**
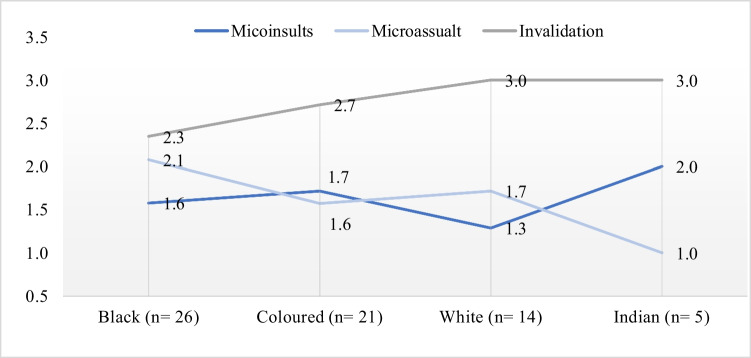
Participants’ ranking of dimensions of microaggression by racial identity (*n* = 66)

As presented in Fig. 3, a general overview of results shows that invalidation was considered most detrimental to wellbeing by all four racial groups of South Africa. On the one hand, microinsults were reported to have the least impact effect on Black and White South Africans’ wellbeing outcomes. Conversely, Coloured and Indian South Africans ranked microassault as having the least impact on their subjective evaluation of wellbeing.

### Racial Differences in the Perceived Effect of Microaggression and Its Dimensions and Manifestations on Wellbeing

#### Black South Africans

In this order, invalidation, microassault, and microinsult were rated to have the most detrimental impact on the wellbeing of Black South Africans. Notable aspects of wellbeing associated with the microaggressions were physical and psychological health, socioeconomic performance, social relationships, and the ability to meet basic needs. Invalidation experiences were reported by Blacks to have the most negative impact on their wellbeing. Such experiences were argued to trivialise the unique experience and history of the Black race and normalise the perception of Blacks as inferior in South Africa. This is exemplified by statements such as, “…such action will disturb me a lot. It makes me feel small and will affect my psychological health.”(NWBLK09); “… this kind of behaviour (that undermines Black culture and experience) facilitates exclusion and is obviously offensive.” (WCBLK01).

Furthermore, across the three manifestations of the three dimensions of microaggression, systemic microaggression emerged as the most important to wellbeing. On the other hand, behavioural and verbal manifestations are tied as the next most important in their negative effect on wellbeing. Systemic invalidation was associated with normalised actions undermining Black South African experience and culture. The weight given to systemic microaggression as the most important of the three manifestations of microaggression can be inferred from the statements of some of the participants. For example, in response to what effect a microassault that manifests as systemic would have on wellbeing, NWBLK03: “I sometimes do fear for my career. When I experience or witness a systemic assault on my race at work, I stand up against it…my manager will always say to me, sometimes you just need to keep quiet; you need to not have an opinion… and that’s something that I fear and affect my mental health…”. Similarly, participants responded to systemic microinsults with statements such as WCBLK04: “Well, for me, as a Black person, I think a lot of Black people, when they see a White or Indian person, they immediately want to be submissive…this is because of the stereotype that Blacks are always less and that is very irritating because it means Blacks are not recognised.” GPBLK05 “… this kind of experience affects my productivity and how much I can contribute to work, family and society.” Likewise, responses to systemic invalidation include the following: WCBLK03 suggests that such actions also affect social relationships. “Even the socially acceptable discrimination still makes me feel undermined. I would definitely feel undermined.” The participants suggest that subtle behaviour that normalises negative stereotypes on a societal scale has strong negative consequences for wellbeing.

**Fig. 4:**
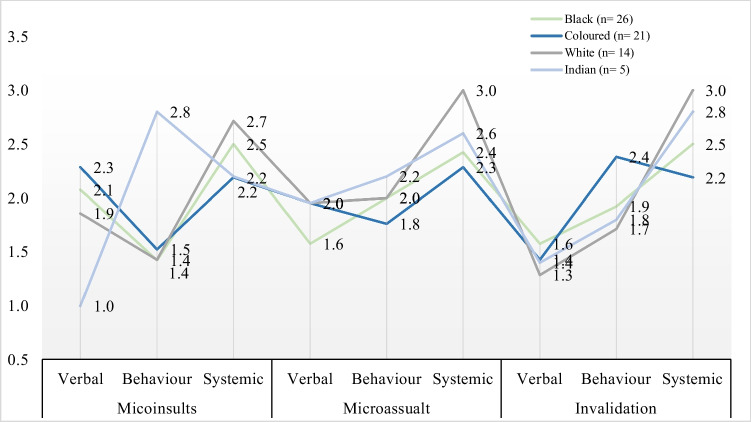
Participants’ ranking of dimensions and the manifestation of microaggression by racial identity (*n* = 66)

On the other hand, participants were split on how behavioural and verbal microaggressions affect their wellbeing. For example, in response to the behavioural assault, GJBLK05: “…this is different from just words. Such behaviour undermines me and will affect my wellbeing, sense of inclusion in the social environment, mastery and self-esteem.” In response to behavioural microinsults, NWBLK06 “…I don’t think that South Africans are at the point where how white people behave or feel about you affect your life. They can be racist towards you; we are used to this kind of behaviour. When it happens, we move!” For verbal microinsult, a participant responded NWBLK06, “Anything that destabilises you and makes you feel down or angry impacts your physical or mental health. And also, it’s beyond just that temporary feeling of being angry or upset. But it subconsciously affects your future interactions, or maybe build your own negative stereotype around people of other race.” Yet another participant’s response to verbal microinsult is that: WCBLK01, “I won’t be angry or upset because I know the person didn’t intend to insult me, but I would address it. I would definitely want to know where you are coming from and why you have stereotypes about my group” In response to verbal assault: NWBLK06: “This kind of verbal assault will not affect my physical health or socioeconomic status since I know that was the intention, but it will definitely affect my mental health and social relationships…” These responses show that behavioural and verbal microaggressions may have a differential impact on the wellbeing of Black South Africans (see Fig. 4).

#### Coloured South Africans

Invalidation had the highest ranking as the most unfavourable for wellbeing. This was followed by microinsult and then microassault. The aspects of wellbeing for which microaggression was consequential for this group were primarily basic needs, socioeconomic performance, social relationships, and psychological health.

The overwhelming unfavourable effect of invalidation is captured in the participants’ statements. For example, GJCMR04 “…such behaviour affects my ability to perform my job more effectively, which affects my income and other aspects of my life.” or WCCMR01, “I’ll feel offended or be upset. Behaviour like this is purely exclusion. It will affect my ability to improve myself. So now I feel that you’re better than me, which then, in turn, makes me upset.”

Further, across the three manifestations of microaggression, the systemic manifestation emerged with the highest ranking for its unfavourable effect on wellbeing. Verbal and behavioural manifestations were tied in the weight of their effects on microaggression. The impact of systemic microaggression on wellbeing is showcased in statements such as KZNCMR04, “as Coloured, we can do nothing about such things. It upset me, but we do not have power in this country. So it sort of helps us to put people in a box. So you know, those people when you go there, you know, you put your guard up, you protect yourself….”

Similarly, KZNCMR03 added that these experiences are responsible for the generally disadvantaged position of the Coloured racial group in South Africa. “It would affect me because I know that we coloured are marginalised because of this… a lot of children are educated but still can`t get a job. So it will bother me and make me angry.” The similar weight that emerged for the effect of verbal and behavioural manifestations of microaggression can be linked to the different reactions participants showed to the same manifestation of microaggression. For example, in response to the effect of verbal microassault on wellbeing: GJCMR01:“… it [authors note: comments or questions based on a negative stereotype about Coloured] does hurt. The accumulated insult we [authors note: Coloured South African] experience creates a barrier to many aspects of life….” Similarly, KZNCMR04, “Yes, they might be complimenting me, but they are insulting my race… I will be offended, which will affect my emotion because it will make me angry.”

On the contrary, GPBLK03: “Coloured people are used to hearing things like that. It is mediocre and doesn’t really affect me that much.” Similarly, participants responded to the effect of behavioural microaggression in the same divided way. WCCMR02: “I find this kind of behaviour irritating. The idea that Coloured people should be a certain way is offensive” or GPCMR01 “So I will feel offended if it’s a behaviour that someone tried to put me at a disadvantage because of your racial group. But it’s easier to address this behaviour.”

#### White South Africans

Consistent with previous racial groups, for White South Africans, experiences of invalidation pulled the most weight as significant for poor wellbeing, followed by microassault and microinsult. The Whites emphasised psychological wellbeing, socioeconomic status, spirituality, social relationships, and emotional wellbeing as core aspects of wellbeing affected by the experiences of microaggression. Participants emphasised the significance of invalidation experiences to their wellbeing through statements such as “…this kind of statement creates insecurity. I feel under economic attack; I feel that I will be attacked. I feel like I will lose my house, possessions, and children just because of your race.” (WCWHT02) or “…this kind of behaviour makes me feel marginalised or undermined based on my racial identity, which affects every aspect of my life” (NWWHT02).

Additionally, a systemic manifestation of microaggression on average across the three dimensions of microaggression was more impactful in producing poor wellbeing. This was followed by verbal and then behavioural manifestations. However, verbal and behavioural manifestations had very similar effects on wellbeing. The stronger effect of systemic manifestation is exemplified by the following statements: “These systemic acts often lead to economic disadvantage and deprive me of opportunities. It is extremely offensive, and it undermines me as a person and breaks me down emotionally and mentally.” (GPWHT04) and “…regardless of the intention, I feel like there are so many common assumptions about White South Africans these days. For example, you’re born a racist because you’re born Whites. Generalising or making an assumption is emotionally damaging and creates social gaps.”(NWWHT01).

Furthermore, the greater weight attached to verbal manifestations of microaggression over behavioural can be found in the following statements: For example, NWWHT03 mentioned that such verbal exchange creates insecurity linked with adverse life outcomes “…so the fact that someone is putting me in a position to feel insecure will negatively affect my life and my work.”; NWWHT04 “I find this kind of statement amusing for the moment. The more I think about it, the more it upset me”; NWWHT04 “I react to this behaviour because if I don’t give attention to it, people will continue to repeat this behaviour.”; NWWHT04 suggests that when such behaviour happens repeatedly, it becomes harder to ignore them and might affect social relationships “if it consistently happens, I feel insulted, and it becomes harder for me not to generalise….” and “… once these things get to you, you eventually build walls that might impact your relationship with certain people.”

#### Indian South Africans

For the Indian South Africans, the invalidation category of microaggression was reported as the most negatively consequential for wellbeing. In turn, microinsult was rated higher than microassault. The aspects of wellbeing affected by microaggression for Indians were social inclusion, socioeconomic status, emotional wellbeing, family relationship, psychological health, and basic needs. Statements which show the overarching effect of invalidation above the other dimensions are captured in the following excerpts: GPIND02 “…it makes me feel bad. These comments undermine me and might also affect the opportunities I get to make money and feed my family”; KZNIND01 “…behaving in a way that disregards my personal identities doesn’t put me in a win situation. It reduces my economic chance because of my race.”

The predominant effect of systemic microaggressions can be gleaned from the reports made by the participants. KZNIND03 “…on a personal level, I feel disappointed because I think people should know better. This is why I avoid certain people”; GPIND01 “systemic invalidation will probably affect many areas of my life. For example, my career will affect the opportunities I’m offered and will stress me a lot. It will also affect how I relate with others or what I expect from them.”

Relative to other groups, behavioural manifestations of microaggression had a stronger effect on Indians. Participants suggest that the exclusion of Indians in South Africa makes it difficult to ignore behaviours based on negative racial stereotypes. GPIND02 “I am more sensitive to what people do than what they say. This kind of behaviour determines if I am in control of what is going on around me. So, when it’s negative, it affects my emotion, relationship with others, and ability to meet my daily need.” Behavioural microassault was associated with an increased effect on psychological health and socioeconomic status GPIND01, “I feel tremendously insulted. It is very hard to relate how such experience affects my psychological wellbeing as Indian South Africa, we are hardly recognised in this country South Africa.”

The relative low effect of verbal manifestations of microaggression on wellbeing is corroborated by excerpts from the transcript. Participants argued that comments or questions based on stereotypes but with no intention to hurt have limited implications for wellbeing. KZNIND02 “Yeah, obviously, these comments affect my mood, but I don’t let it affect my relationship with others or other parts of my life.”

## Discussion

The findings of this study show that the experiences of microaggression in its different dimensions and manifestations negatively impact the wellbeing of the South African sample included in this research. Through a qualitative approach using FGDs, participants narrated their experiences of microaggression, its impact on their wellbeing, and, more importantly, how their racial identity determines their life outcomes.

Microaggression, the subtle everyday discrimination, indignities, and insults encountered as a result of the social group to which an individual belongs were confirmed as prevalent in a sample of South Africans. The three dimensions of microaggression — microinsult, microassault, and invalidation — were reported to have differential effects on wellbeing. The findings of this study confirm that in multi-group settings, the interaction between constituent groups could have implications for the wellbeing of individual members of each group. Participants of all racial groups narrated direct and indirect encounters of microaggression at work, school, and generally from members of society. Of the three dimensions of microaggression, participants reported invalidation as the most consequential for wellbeing.

Invalidation was described as experiences involving encounters with others that trivialise or belittle one’s racial group and identity. Its unique experiences were argued to affect the quality of wellbeing of individuals and an entire racial group on a large scale. Existing literature supports the link between the experience of invalidation and wellbeing. Invalidation experiences in various forms have been linked to maladjustment, depression, poor self-esteem, and other adverse mental health outcomes [7, 19]. The overarching impact of invalidation for wellbeing for all racial groups above the other dimensions of microaggression is perhaps due to the centrality of racial identity in defining life outcomes for the South African participants. In apartheid South Africa, citizenship and human rights were determined based on racial identity. This resulted in some racial groups having more rights and privileges over others [36]. The experience of apartheid lives on in the memory of South Africans and forms the basis of distrust and aversion to social relations with racial outgroups [25, 37]. Each racial group in our study perceive their experiences during apartheid as unique and peculiar. Participants from groups that experienced the brunt of discrimination and subjugation, such as the Blacks and the Coloureds, perceive that there has not been much improvement in their conditions. When asked how well they can achieve the things important to their wellbeing relative to other racial groups, Blacks and Coloured perceived themselves to still be relatively disadvantaged. Whites and Indians, who are relatively economically well-off, reported that their social position makes them targets of discrimination and exclusion. Thus, all racial groups perceive past and current invalidation of their racial identities and experiences and what these identities and experiences imply for their life outcomes.

The systemic form or manifestation of microaggression across all racial groups (except Indians) was reported to have the most impact on wellbeing in our study. Experiences of microaggression embedded in societal structure and processes were more detrimental to wellbeing. This is because such experiences frequently occur, raise the racial identity consciousness of the individual, and are consequential to survival. Occurrences that directly denigrate one’s social identity can profoundly affect an individual life outcome [47]. When people live in an environment where their ingroup and identity are mocked, relegated, and denied access to resources, it can create a perpetual state of threat consciousness [27]. Participants in the current study reported long-standing inequalities and discrimination in the workplace, access to healthcare, food and nutrition, and basic human dignity as forms of systemic microaggressions. In South Africa, systemic experiences of microaggressions may be reminders of the systemic oppression and discrimination of apartheid for some individuals. For instance, as reported by participants, all other things being equal, racial identity is still considered an influential factor in the creditworthiness of an individual in applying for loans or other credit schemes. Also, participants perceived affirmative actions to reduce economic disparities for some racial groups as unfairly limiting the opportunities available to others. For example, Coloureds and Whites reported that the Broad-Based Black Economic Empowerment (BBBEE) and other legislative tools prevent them from exploring and securing opportunities in the environment. For some of these participants, when they have experienced discrimination based on some form of affirmative action, such occasions were perceived as systemic invalidation experiences.

Furthermore, emotional wellbeing, psychological health, and socioeconomic status were the most highlighted aspects of wellbeing affected by microaggression across all racial groups. Emotional wellbeing comprises having positive emotions, feelings, and thoughts and coping with discomfort and stressful situations. Psychological health for participants meant good mental health. Socioeconomic status encompasses concerns about having enough money, a stable job, and advancement in education. Participants reported that experiences of microaggression affect their ability to be happy, be in a positive state of mind and have access to the socioeconomic resources to fulfil their basic needs and provide for their families. However, while all racial groups emphasised these wellbeing factors as the most affected when they experience a microaggression, Blacks and Coloureds reported greater socioeconomic concerns than Whites and Indians. The greater weight attached to socioeconomic factors for these groups corresponds with the current social inequality experienced by people of colour in South Africa. Blacks and Coloureds post-apartheid have experienced growing inequality in employment, education, and access to healthcare [20, 45]. These perhaps account for why socioeconomic concern was the most important for the wellbeing of these groups.

The findings from the current study provide insights into microaggression outside of western countries’ contexts. It provides a base for comparing how microaggression is experienced in different settings. On the other hand, it is essential to note that reference to “racial identity” can also be understood as microaggression and reproduce microaggression. However, addressing social and economic inequities requires exploring the different identities and identifying the disadvantaged groups within a population.

### Limitations and Strength of the Study

As shown from the findings of this study, the effect that the experience of microaggression has on wellbeing is subjective, and the sensitivity to such experiences varies across individuals, racial groups, and the specific nature of the experience. As such, exploring dimensions and manifestations of microaggression based on their effects on wellbeing is limited in its generalisability across individuals. However, the similarities in microaggression experiences within racial groups and the near consensus on which dimensions have the most effect on wellbeing validate our exploration of the different dimensions and manifestations and their impact on wellbeing.

Generalising results from the phenomenological approach is contentious [43] partly due to this approach’s exploratory nature and its homogenous sample. Nevertheless, the current study drew its sample from different individuals across provinces in South Africa using purposeful sampling to ensure representation of varying demographics and socioeconomic backgrounds. Therefore, the interpretation of the result is based on the assumption that individuals from the same racial groups share similar experiences. Future explorations of this topic should consider approaches that allow for a more comprehensive interpretation of research results.

Finally, public knowledge of the different dimensions and manifestations of microaggression remains limited. As such, for the current research, it was essential to share situational examples to help participants distinguish between the various forms of microaggression. This approach, however, risks that participants base their responses on a specific example rather than the concept behind the form. To address the potential bias, the moderators repeatedly encourage participants to look beyond the example while discussing each form. Further research should establish clear and simple definitions for the different forms to ease the exploration of microaggression in health and humanitarian context.

## Conclusion and Implication of Findings

The outcome of this study projects a wide spread of perceived microaggression among the participants. Secondly, it confirms that microaggression is experienced by both the racially minoritised and dominant groups in South Africa. Third, participants’ narrative of their experiences of microaggression confirms that microaggressions are reflective of racism. Fourth, findings confirm the association between experiences of microaggression and wellbeing for South Africans. The harmful impact of microaggressions was captured in how participants narrated their experiences. This impact is also related to the historical context of apartheid in South Africa, in which there was social segregation and racism. People of all racial groups differ in the weight attached to experiencing the different dimensions of microaggression.

Nevertheless, they shared a similar understanding of when a racial microaggression occurred and its impact on their wellbeing. This, therefore, implies that addressing microaggression in South Africa is vital for improving the welfare of the citizens. Wellbeing is a public health variable that can be used as an index of the social development of a society. It is closely linked to positive health outcomes and productivity and is affected by microaggression. Results from the current study collaborate with previous results to confirm that experiencing discrimination in the form of microaggression is associated with poorer wellbeing [15, 29, 32, 40].

The results confirm that the environment exerts a lot of effect on the self-reported wellbeing of citizens [18, 38]. Similarly, living in a society where there are everyday slights, insults, and discrimination can greatly impact how people feel secure in their identity and their connectedness to it [52]. Group connectedness provides stability and certainty in multi-group societies due to the group rivalry that pervades such societies. Indeed, the wellbeing of the social group can be closely tied to the wellbeing felt by individual members. While extant laws explicitly address discrimination, it is vital to take into cognisance for policy and regulation purposes the subtle actions that also communicate hostility and denigration to people based on their identity. For research, further exploration of microaggression as a predictor of life outcomes is paramount.

## Supplementary Information

Below is the link to the electronic supplementary material.

Supplementary file1 (DOCX 24 KB)

## Data Availability

Data included in this report are available on request.
