# Supplementary material for: Interpreting Microaggression as a Determinant of Wellbeing
Source: J Racial Ethn Health Disparities. 2022 Oct 12;10(5):2470–81. doi: 10.1007/s40615-022-01426-z (PMC10482806; doi:10.1007/s40615-022-01426-z)
Supplement: Supplementary file 1 — Supplementary file1 (DOCX 24 KB) [file 40615_2022_1426_MOESM1_ESM.docx]

**Appendix 1: Examples of categories and the manifestation of microaggression**

**Microaggression** is a form of discrimination via “[insensitive](https://www.wordhippo.com/what-is/another-word-for/insensitive.html)” or/and “unspoken” bias. Microaggression can be comments or questions (Verbal) or behaviours (Behavioural) that are hurtful, stigmatising, or discriminatory to a particular marginalised group. It can also be in the form of subtle discrimination within society **(**Systemic**)**.

| **Types/Categories of microaggression** | **Dimensions** | **Examples** |
| --- | --- | --- |
| **Microinsult:** Unintentionally behaves in a discriminatory manner | Verbal | - Oh, you are Coloured/Black! But you seem so educated! - Oh, you are White/Indian/Coloured! But you are not racist! |
|  | Behaviour | - Someone approaches you to ask where he can buy Cocaine, with the presumption that Whites are drug dealers - Someone approaches you to ask where he can buy weed, with the presumption that Blacks are drug dealers - A White person clutches their purse as you, a Black man approaches |
|  | Systemic | - You got into an argument with someone of a different race. A police officer stops and goes straight to the other person and asks if he/she is okay (there is a general assumption – stereotype – that people from your group are usually aggressive) |
| **Microassault:** Conscious-direct insults that communicate that the targeted party is of lesser worth | Verbal | - People of your racial identity are racist, arrogant, and annoying. - People of your racial identity are rude, loud, and annoying |
|  | Behaviour | - Touches your hair; White hairs are strange - Touches your hair; black hairs are sticky - You enter a store, and the store owner or attendant follows you to monitor you because “people of your racial group usually steal.” |
|  | Systemic | - A (White) & B (Indian) are qualified for a Job. B got the job because Indians are considered better at IT - You are denied a loan because the banking system considers Blacks as not reliable and, therefore, too risky |
| **Invalidation:** Undermine or ignore the experiences of a certain group of people | Verbal | - Someone told you that you cannot be South African because your skin colour is different (too white) - Someone making jest of your traditional or religious attire - Someone of a different racial group from you finds your name too hard to pronounce and then shortens your name or calls you by something else |
|  | Behaviour | - You are the only one from a different racial group in a community meeting. The other participants started speaking a language you do not understand, therefore, making it difficult for you to participate |
|  | Systemic | - Your colleague from a different race is asked to present an idea you have been working on because it is considered more sellable coming from someone of a different race |

**Important Note:** The experiences of microaggression may vary across racial groups based on the stereotypes associated with each racial group feature. Therefore, the above examples were rephrased or used based on their applicability to the different racial groups. In addition, participants also gave personal examples of their experiences of microaggression. Participants were encouraged to think beyond the specific examples and rate their experiences of microaggression based on the concept definition and its effect on their wellbeing.
